# Perspectives of practitioners on support for caregivers of children with intellectual disability

**DOI:** 10.4102/curationis.v47i1.2559

**Published:** 2024-08-30

**Authors:** Lebogang L. Molefe, Leepile A. Sehularo, Daleen M. Koen

**Affiliations:** 1 Quality in Nursing and Midwifery (NuMIQ) Research Focus Area, Faculty of Health Sciences, North-West University, Potchefstroom, South Africa; 2 Lifestyle Diseases Research Focus Area, Faculty of Health Sciences, North-West University, Mahikeng, South Africa

**Keywords:** children, mental health, practitioners, profound intellectual disability, perspectives, secondary caregivers, support

## Abstract

**Background:**

Children with profound intellectual disabilities are unable to do anything for themselves and require full-time care in healthcare facilities. While caring for children, secondary caregivers become overwhelmed. Coupled with little or no support, the overwhelming work affects their psychological, social and financial well-being. Mental healthcare practitioners have perspectives on conditions under which secondary caregivers work and how can they be supported. Little is known about such perspectives.

**Objectives:**

This study aims to explore and describe the perspectives of mental healthcare practitioners regarding the conditions under which secondary caregivers of children with profound intellectual disabilities work and how can they be supported.

**Method:**

A qualitative-exploratory-descriptive and contextual research design was adopted using a non-probability purposive sampling technique. This study was conducted in Gauteng province. Semi-structured individual interviews were performed to collect data. Content data analysis and ATLAS.ti were used to analyse the data.

**Results:**

Knowledge and skills development, stress reduction, resources and motivation were themes that emerged.

**Conclusion:**

This study explored and described the perspectives of mental healthcare practitioners regarding the conditions under which secondary caregivers of children with profound intellectual disabilities work and how can they be supported. If implemented, perspectives can improve the holistic well-being of secondary caregivers.

**Contribution:**

This study broadened an understanding of how secondary caregivers can be supported. Future researchers can use study results to develop programmes, intervention strategies and frameworks to support secondary caregivers.

## Introduction

Children with profound intellectual disability cannot live independently and require supervision and help with self-care activities. They are affected by a combination of cognitive disability and neuromotor and sensory impairments (Liesbeth & Goossensen 2023:1). Their high level of dependency has serious implications for them and their secondary caregivers. The children are often admitted to healthcare facilities because families are unable to cope with the burden of caring, leaving the burden to secondary caregivers. Secondary caregivers experience psychological, emotional, social and financial challenges because of caring for children with profound intellectual disabilities (Fernández-Ávalos et al. 2020:3). According to a study by Ogunfolaju (2020:158), intellectual disability is defined by the American Association on Intellectual and Developmental Disabilities (AAIDD) as a significant sub-average intellectual functioning associated with concurrent impairments in adaptive behaviour manifested during the developmental period. Children’s Act of South Africa (2005) described a child as a person under the age of 18 years. According to the *Diagnostic and Statistical Manual of Mental Disorders (DSM-5*^*TH*^), a child with a profound disability has an intelligence quotient (IQ) of below 20 or 25 (Kent, Burgess & Kilbey 2018:67) and hence needs high-intensity and pervasive support across all environments. Such a child has significant limitations in self-care, continence, communication and mobility, and such a child needs complete custodial or nursing care and is total care dependent (Patel et al. 2020:2281). Based on the definitions, a child with a profound intellectual disability is someone below the age of 18 years, who depends on someone to take care of him or her to ensure that his or her basic human needs are completely met, in this case, a secondary caregiver.

Secondary caregivers are people employed in healthcare facilities, whose responsibilities are to assist children with intellectual disabilities in activities of daily living, such as bathing, dressing, feeding, changing nappies, playing with a child, providing stimulation, giving medication and talking to a doctor on behalf of a child (Larkin, Henwood & Milne 2019:59; Mollica et al. 2017:4483). Secondary caregivers of the study are care workers, auxiliary nurses, enrolled nurses and newly qualified registered nurses employed in mental healthcare institutions admitting children with profound intellectual disabilities. Auxiliary nurses, enrolled nurses and registered nurses have undergone professional training for the job in various nursing education institutions licensed to offer such training. Their training and registration are controlled by the South African Nursing Council (SANC). Care workers would have a 6-month or 1-year certificate of training from private schools before employment. Some might not have such a certificate during the employment date; however, an employer would send the person for training prior commencement of a job. These categories of staff are employed to provide care to children admitted and work various shifts. Caring for admitted children is a 24-a-day work, compelling staff to relieve each other, meaning that when others knock off, others will resume duty. Activities involved in caring include bathing, dressing, feeding, changing nappies, playing with children, providing stimulations and giving children medication (Larkin et al. 2019:58). The caregiving role is hectic, complex, demanding and overwhelming as it involves countless roles and responsibilities, and hence, support for secondary caregivers is required. The required support should be from families of children, the community at large and, most importantly, the multidisciplinary team members. A lack of such support can result in psychological, emotional, social and financial challenges (Fernández-Ávalos et al. 2020:3).

The multidisciplinary team members of the study are mental healthcare practitioners who provide specialised care to children with profound intellectual disabilities. They include specialists such as a speech therapist who stimulates swallowing reflexes and teaches the child how to respond when spoken to as children are unable to speak, a physiotherapist who provides passive exercises to prevent contractures and a psychologist who attends to the child’s psychological being. They regularly see children in the wards and spend a maximum of 2 h with children to provide the service. When in the wards, they interact with caregivers to receive updates on the progress of children, because caregivers are the ones who spend 24 h a day with children. During interaction and observations of non-verbal communication cues from caregivers, mental healthcare practitioners encountered caregivers’ feelings. Little is known about the perspectives of mental healthcare practitioners regarding how secondary caregivers of children with profound intellectual disabilities can be supported because no study has reported on the perspectives of mental healthcare practitioners regarding the support required by secondary caregivers, hence the study. Mental healthcare practitioners are referred to as social workers, psychologists, counsellors, psychotherapists, psychiatrists, physiotherapists, occupational therapists, medical practitioners, nurses and speech therapists (Mental Health Care Act 2002). It is necessary to understand the perspectives of mental healthcare practitioners regarding how secondary caregivers can be supported because without support, they are at risk of clinical depression and challenges associated with lack of support (Mbugua et al. 2021:2). A lack of support can further compromise secondary caregivers’ life satisfaction and lower their quality of life, thus reducing their life satisfaction (Gebeyehu, Sahile & Ayalew 2019:4). With support, secondary caregivers may be able to develop coping strategies for dealing with stress and will further develop resilience (Dada, Bastable & Halder 2020).

### Purpose of the study

This study aims to explore and describe the perspectives of mental healthcare practitioners regarding the conditions under which secondary caregivers of children with profound intellectual disabilities work and how they can be supported.

## Research methods and design

### Study design

A qualitative, exploratory, descriptive and contextual research design was used. The design assisted the authors in having a better understanding of the conditions secondary care workers work under and how they can be supported. It further assisted the researcher in exploring, discovering, uncovering, gathering information, describing and summarising the data gathered and the results. It produced unquantifiable data using open-ended questions (Asenahabi 2019:77).

### Setting

The study was conducted in Gauteng province, South Africa. This province was chosen because of its highest population, highest number of secondary caregivers and highest number of children with profound intellectual disabilities. Gauteng province has a population of 15 099 422 in comparison to other provinces such as KwaZulu-Natal with 12 423 907, Western Cape with 7 433 019, Eastern Cape with 7 230 204, Limpopo with 6 572 720, Mpumalanga with 5 143 324, North West with 3 804 548, Free State with 2 964 412 and Northern Cape with 1 355 946 (Stats SA 2023:52). About 6.4% of children in Gauteng province have profound intellectual disability (Stats SA 2014:25). Although Gauteng province is not the highest in children with profound intellectual disability, competition for scarce health care resources (human resources, health care equipment and material resources) is high. The authors sought to include mental healthcare institutions with the highest number of children and secondary caregivers. Collaborating with the Gauteng Department of Health, the authors obtained a list of the facilities that have a higher number of children with profound intellectual disabilities, hence the four facilities of the study. Each metropolitan municipality was covered.

### Study population and sampling strategy

A non-probability purposive sampling technique was used. Participants were selected because they had characteristics that the authors needed. The study population was mental healthcare practitioners in Gauteng province, within four selected mental healthcare institutions, in the specific wards that admitted children with profound intellectual disabilities. Twenty-eight mental healthcare practitioners were approached and requested to participate. Twelve practitioners agreed, others declined the invitation, citing their busy schedules and not feeling comfortable to participate. Their decisions were respected. To confirm eligibility for participation, principles of inclusion and exclusion criteria were followed. Hence, only mental healthcare practitioners who had experience of at least 5 years and above in a profession and had given voluntary consent to participate were included because they provided much richly needed information. All other healthcare practitioners were excluded.

### Data collection

Unstructured interviews were used to collect data. Questions asked were not prepared in advance but arose spontaneously in a free-flowing conversation. Different participants were asked different questions. The questions posed assisted the authors in understanding the conditions under which secondary caregivers of children with profound intellectual disabilities work, including how they can be supported. Interviews were conducted at the participants’ offices, at the times suitable to participants. Participants decided on the date and time of the meeting. Interviews occurred during March and April 2022 and lasted between 20 and 45 min. Table 1 provide an overview of the demographic information of participants. The biographic information was necessary because the researcher needed to be sure that participants were over 18 years old to give voluntary consent to participate, that participants had the experience of at least 5 years and above in a profession and that participants were inclusive of all genders. Participants were three psychologists, three social workers, one auxiliary social worker, two physiotherapists, two occupational therapists and one psychotherapist.

**TABLE 1:** Participants’ information.

Participant	Gender	Age (Years)	Category of a profession	Years of experience
A	Female	33	Psychologist	10
B	Female	28	Social worker	7
C	Female	30	Auxiliary social worker	6
D	Male	22	Physiotherapist	9
E	Female	41	Psychotherapist	15
F	Male	36	Occupational therapist	12
G	Female	25	Psychologist	10
H	Male	31	Social worker	13
I	Female	45	Occupational therapist	11
J	Female	34	Physiotherapist	14
K	Male	37	Psychologist	6
L	Female	28	Social worker	8

The following questions were asked to all participants: ‘What are your perspectives regarding how secondary caregivers of children with profound intellectual disabilities can be supported?’ Clarity-seeking questions were ‘What do you mean, please clarify, please explain further’. The researcher listened to responses, noticed non-verbal cues and took field notes. Data were collected until data saturation was reached, that is, until no new information emerged, which occurred on participant number eight. However, all 12 participants were interviewed to confirm if saturation did indeed occur. All interviews were audio-recorded with the consent of participants.

### Data analysis

Data were analysed using content analysis and ATLAS.ti. Content analysis is a technique used to determine the presence of certain words, themes or concepts within the data to explain a phenomenon (Polit & Beck 2018:387). Both the first author and a co-coder independently analysed the data using the same format. Creswell’s six steps were followed to analyse the data (Creswell 2013:182). The audio tape recorder was listened to and data were transcribed. Each transcript was performed separately. Transcripts were read and reread. Information on the transcripts were fed into the computer. This activity was carried out to enable the computer to assist in categorising data to create and assign codes, using ATLAS.ti software. Codes were reviewed and revised by the first author to ensure that identified concepts are of importance to the theory of the research project. Codes were finally converted into themes and sub-themes. Themes and sub-themes were presented cohesively. A meeting was convened between the researcher and a co-coder to discuss and verify identified themes and sub-themes. Where there were disagreements, a thorough discussion was held and consensus was reached on the final themes and sub-themes.

### Trustworthiness

Trustworthiness or rigour is a degree of confidence in data, interpretation and methods used to ensure the quality of a study (Polit & Beck 2018:335). Five testing criteria were observed: credibility, dependability, confirmability, transferability and authenticity (Polit & Beck 2018:336). Credibility was attained by verbatim transcription of interviews, the use of a co-coder during data analysis (a co-coder assisted because the researcher viewed some data differently through the lens of her knowledge and experience and the co-coder assisted to prevent the lens from obscuring participants’ experiences and meanings) and triangulation (data from different sources [participants], data from different times, data from different people, in different places [different mental health care institutions] participants were combined), by using semi-structured individual interviews, as well as taking field notes. Dependability was attained through the description and application of the research methodology, audit trial (the researcher was transparent with steps taken from the start to the end and the findings thereof) and the use of a co-coder during data analysis to verify the findings. Confirmability was attained through ensuring that the findings were not shaped by the researcher’s bias, motivation or interest, and hence, the researchers ensured that the data represent the information that participants provided. Transferability was attained through the thick description of the research findings from multiple sources of data collection, that is, from multiple mental healthcare practitioners. The study will be published with sufficient data to enable readers to conclude whether transferability can be possible. Authenticity was attained by ensuring that the report conveys perceptions of mental healthcare practitioners, nothing more.

### Ethical considerations

Ethical clearance was granted by the Health Research Ethics Committee (HREC) of North-West University (reference number NWU-00462-20-A1). Furthermore, permission to access the mental health care institutions was granted by the Director of Gauteng provincial Department of Health (GDoH), as well as the Chief Executive Officers (CEOs) of the four mental health care institutions. The managers of the institutions further gave permission for the interviews to proceed. Participants gave voluntary consent to participate and agreed to be audio-recorded. Participants were reassured that the recorded information would never be shared with anyone without their consent. All participants were conscious and fully aware of their participation contracts. Participants’ names were not used, but codes were assigned, for example, P-A would indicate participant A.

## Results

Table 2 gives a summary of themes and sub-themes that emerged and are further explained.

**TABLE 2:** Themes and sub-themes.

Themes		Sub-themes
1.	Knowledge and skills development	1.1. Regular training 1.2. Demonstration
2.	Stress reduction	2.1. Emotional support 2.2. Regular counselling 2.3. Group therapies 2.4. Parents’ involvement
3.	Resources	3.1. Medical equipment 3.2. Human resources
4.	Motivation	4.1. Appreciation and reward 4.2. Incentives

### Theme 1: Knowledge and skills development

Knowledge is the understanding, awareness and retention of information, and concepts acquired through perception, learning and reasoning, an organisational culture, skills, reputation, intuition and codified theory that influences human behaviour and thought (Abubakar et al. 2019:108). A skill is a learned power of doing something competently (Brown & Neve 2023:12). The two sub-themes for knowledge and skills development are regular training and demonstrations.

#### Regular training

Training refers to short-term training courses designed to give employees additional knowledge, and practice skills, and improve their work performance (Himam 2022:8). Participants said it appears that secondary caregivers such as auxiliary nurses, enrolled nurses and care workers are not adequately skilled in their limited training to provide care to children with profound intellectual disabilities and lack adequate knowledge regarding profound intellectual disability. Their training curriculum does not cover intellectual disability and does not allow training on intellectual disabilities. Registered or professional nurses have undergone professional training on intellectual disabilities. However, there is a need for regular in-service training on intellectual disabilities to upskill them on the latest developments in caring for children with profound intellectual disabilities. Caregivers cited that intellectual disability is a speciality, and therefore, a person who is not trained on intellectual disability, or who has not worked at intellectual disability facilities for a longer period, needs regular training to improve knowledge and skill and to be able to provide quality care:

‘Secondary caregivers did not do a module on intellectual disability as a speciality and therefore find it difficult to care for children.’ (P-A)

‘Managers must acknowledge the fact that there is a gap in caregivers’ knowledge of intellectual disability and must provide regular training for caregivers in the form of in-service training.’ (P-D)

‘Regular in-service training can better caregivers’ knowledge regarding intellectual disability.’ (P-I)

Training can still be performed in the form of in-service training, as cited by another participant:

‘There is no need to send them for full-time training, but on-site training, an in-service training will be sufficient.’ (P-J)

#### Demonstration

Demonstration is a method of teaching by demonstrating goods, events or teaching indirectly through the use of certain media in accordance with the subject or the material being prepared. The method allows students to observe what is shown during the lesson and later practice it correctly (Aisah 2021:3). Participants further cited that secondary caregivers need a demonstration of skills such as bathing, feeding, changing positions, transferring from bed to chair, stimulations and talking to children. According to participants, such demonstration can be done through in-service training:

‘A practical demonstration of skills such as feeding, bathing, stimulating a child, providing exercises, and positioning a child will be beneficial for secondary caregivers!’ (P-E)

‘Practical demonstrations can improve caregivers’ confidence in the provision of safe and quality care.’ (P-F)

‘Some caregivers just lift the children because they do not have a specialised skill of lifting, they end up hurting their back. A demonstration will therefore equip them with the skill of how to lift correctly.’ (P-I)

Participants have suggested regular training and demonstrations as an ideal solution to improve caregivers’ knowledge and to improve competency in skills performance. Should the suggestions be adopted, a reduction in the problems such as lack of knowledge and incompetency will be the result.

### Theme 2: Stress reduction

Stress is a natural reaction to specific demands and events and is managed or reduced through a range of techniques, strategies and therapies designed to help people control their stress (Scott 2023:1). The four sub-themes for stress reduction are emotional support, regular counselling, group therapies and parents’ involvement.

#### Emotional support

Emotional support is showing care and compassion for another person, and it is a critical way of achieving patient-centred care (Bradshow et al. 2022:4). Participants cited that because of the stressful nature of their work (lifting, transferring from bed to chair, bathing, feeding, changing positions every hour), secondary caregivers require emotional support:

‘Secondary caregivers require a lot of emotional support. The work that they do is exhausting emotionally. Currently, they do not receive support from management.’ (P-H)

‘Managers must be concerned about the well-being of caregivers. It does not take anything away from a manager to always ask questions such as “How are feeling today”; “Are you ok?”; “How can I help?” This will enable a caring manager to identify the emotional support that the caregiver requires.’ (P-E)

‘Management must organise debriefing sessions for caregivers. They need that to relieve stress and exhaustion.’ (P-K)

#### Regular counselling

Counselling is a process that involves a trained counsellor helping individuals to find ways to work through and understand their problems, promote a healthy lifestyle and improve the quality of life and overall health (Kariemlou et al. 2019:350). Participants were advised on regular counselling sessions for secondary caregivers, as well as debriefing sessions as strategies to assist them in managing stress:

‘Secondary caregivers require regular counseling because taking care of children with intellectual disabilities is draining.’ (P-B)

‘If stressed or feeling overwhelmed, caregivers must be referred immediately for counselling.’ (P-G)

‘This job is traumatising. Regular debriefing can assist caregivers a lot.’ (P-K)

#### Group therapies

Group therapy is the management of multiple individuals at once, who have a variety of psychological problems such as emotional trauma, anxiety, stress and depression (Malhotra & Baker 2024:1). Participants advised that secondary caregivers need to have regular group therapies. When in groups, they can become relaxed and be able to vent their fears and frustrations, because they realised that they are not the only ones with challenges:

‘Caregivers must be given time off to go out and have fun as a group of colleagues who work together. When in groups, they can be able to share their frustrations and console each other.’ (P-C)

‘Secondary caregivers must be provided an opportunity to form groups for therapy purposes. In groups, they feel more relaxed, feel not judged when voicing their fears and frustrations, and can derive support from one another.’ (P-G)

‘Group therapies can assist an individual to ventilate. A person can even see that she is not the only one with problems.’ (P-H)

#### Parents’ involvement

Parents are primary caregivers and must be involved in the care of their children during hospitalisation. The family-integrated care embraces the importance of parents in the child’s life, and it involves being present most of the time in the unit and taking over some tasks. This changes the dynamics of the parental role from passive observers to playing an active role in caregiving and making partnerships between parents and healthcare professionals (Van Den Hoogen & Ketelaar 2022:294). Participants cited that parents of children must play a significant role in supporting secondary caregivers in their work by visiting their children regularly and requesting to take their children out for a certain period to allow secondary caregivers to relax and have a rest:

‘Secondary caregivers need to be relieved from the responsibilities of taking care of children on their own. Parents must also assist.’ (P-E)

‘Parents must visit their children often, spend a lot of time with them, and even take them home for a weekend. This will give caregivers a chance to rest and to relax, thus reducing stress and burnout.’ (P-F)

‘Some parents never visited their children since admission, they have dumped children in a hospital, leaving the caregivers to struggle alone. Parents need to take responsibility for assisting with caring.’ (P-I)

Stress reduction is important. Participants recommended the implementation of multiple strategies to reduce stress. If implemented, strategies can assist in the elimination of stressors and effective management of stress.

### Theme 3: Resources

The term resource has been consistently defined as a non-directional physical, psychological, social or organisational characteristic that functions to achieve work goals or reduce demands on the physiological and psychological cost of work (Lee, Rocco & Shuck 2019:10). The two sub-themes for resources are medical equipment and human resources.

#### Medical equipment

Medical equipment is an article, instrument, apparatus or machine used in the prevention, diagnosis, treatment and management of diseases, or for detecting, measuring, restoring, correcting or modifying the structure or function of the body for some health purpose (Zonani et al. 2021:1). Participants cited that secondary caregivers require adequate equipment necessary to provide quality care for children, because insufficient or lack of medical equipment necessary to provide care might lead to distress, and even make secondary caregivers to feel incompetent:

‘Caring for children with an intellectual disability is very hectic, and if there are no supportive tools-of-traits, medical equipment such as assistive devices for lifting, wheelchairs, proper beds, showers designed for children, nappies, it becomes overwhelming.’ (P-C)

‘Secondary caregivers work hard. The least that management can do to support them is to provide them with the medical equipment necessary to provide care. When working on a budget, they need to prioritise medical equipment.’ (P-G)

One participant further emphasised that lack of equipment causes frustrations, burn-outs and lack of confidence in secondary caregivers:

‘Lack of equipment frustrates care providers, namely secondary caregivers. They feel useless, worthless, and stressed. It appears like they do not know what to do, yet the problem is lack of equipment.’ (P-F)

#### Human resources

Human resources in this study refers to adequate staffing. Each institution has a human resource department that is responsible for ensuring adequate staffing, and further focuses on investing employees (hiring, training and supporting employees). The department further focuses on investing in employees, ensuring their safety and managing all aspects of staffing, from hiring to compensation and development. The basic principle is that healthcare providers must have sufficient staff on duty to provide care safely and effectively (Ball & Griffiths 2022:872). Participants emphasised the need for adequate human resources in caring for children with intellectual disabilities:

‘There is a need for adequate staff because it is difficult to care for the children, and now it becomes worse with staff shortage. Management must prioritise employing more caregivers so that the current ones do not become overwhelmed.’ (P-D)

‘It is unfair to expect one secondary caregiver to take care of twenty children. That is the reason why secondary caregivers are forever on sick leaves because they are overwhelmed.’ (P-F)

One participant cited that human resources should include all multidisciplinary teams because caring for children is a team effort, and it cannot be the sole responsibility of secondary caregivers:

‘Caring for children with a profound intellectual disability needs multidisciplinary team members, inclusive of caregivers, physiotherapists, psychologists, social workers, speech therapists, and occupational therapists.’ (P-E)

Participants’ responses describe the severe shortage of multidisciplinary team members who render care to children with profound intellectual disabilities, which is even severe in the category of secondary caregivers. The human resource department must ensure adequate staffing by hiring more caregivers and multidisciplinary team members to ensure the delivery of quality care for children, reduce the burden of caring for caregivers and create a safe and healthy working environment.

### Theme 4: Motivation

Motivation describes why a person does something, the driving force behind human actions. Its process involves initiating, guiding and maintaining goal-oriented behaviours (Cherry 2023:1). Two sub-themes for motivation are appreciation and reward, and incentives.

#### Appreciation and reward

Appreciation is the act of giving someone a proper value. Appreciation is often used interchangeably with gratitude. It is the appreciation of what is valuable and meaningful to oneself. Reward is one form of strategy used to appreciate a person, and it involves recognition of service, effort or achievement (Shi et al. 2022:629). A participant of the study cited that secondary caregivers need to be appreciated for the hard work that they do:

‘Secondary caregivers must be appreciated for the work that they do. They work very hard.’ (P-D)

Two participants further explained how managers can appreciate secondary caregivers:

‘Managers must constantly remind secondary caregivers that they are valuable personnel in the health care setting and must reward them with tokens of appreciation.’ (P-F)

‘When doing performance appraisals, managers should score secondary caregivers much higher so that they can be rewarded. This will boost their morale and self-esteem.’ (P-H)

Appreciating and rewarding secondary caregivers is key. When appreciated and rewarded, caregivers become motivated to soldier on despite the difficulty of the work they are faced with.

#### Incentives

Incentives are things that motivate or encourage a person to do something and can include a payment or a concession to stimulate greater output or investment. Incentives are the reason employees feel energetic and motivated towards their work (Liu & Liu 2021:2). Participants cited that secondary caregivers need to be given incentives as a way to motivate them:

‘Secondary caregivers must be remunerated better. Addition to their basic salaries should be incentives in the form of a once-a-year bonus.’ (P-A)

‘Caregivers must receive medical aid subsidies, and their salaries should not be anything less than ten thousand rands. To be honest, they are entitled to higher salaries and incentives than any other health care professionals, because they work more than any of us here.’ (P-G)

‘Caregivers start working from seven (either morning shift or night shift), and knock off at seven, meaning, they do a twelve-hour shift. They therefore deserve incentives to compensate for their already low salaries.’ (P-I)

Many participants have recommended incentives for caregivers as a strategy to motivate them and to make them feel valued. If adopted, a recommendation will provide a permanent solution to keep caregivers happy at work, thus providing quality care to children.

## Discussion

The objective of this study was to explore and describe the perspectives of mental healthcare practitioners regarding how secondary caregivers of children with profound intellectual disabilities can be supported. Identified themes (e.g. knowledge and skills development, stress reduction, resources and motivation) and sub-themes (e.g. regular training, demonstrations, emotional support, regular counselling, group therapies, parents’ involvement, medical equipment, human resources, appreciation and reward, and incentives) provided insight to how secondary caregivers of children with profound intellectual disabilities can be supported, based on the perspectives of participants. The first theme is knowledge and skills development. Knowledge is a familiarity, awareness and understanding of a fact, information, description or skill. A skill is the ability to carry out a task with an exceptional outcome. It includes putting into action the knowledge gained (Kassema 2019:8). Competency is key in a skill demonstration, which relates to the notion of proficiency and mastery used in a field, the ability to integrate and apply contextually appropriate knowledge, skills and psychosocial factors (such as beliefs, attitudes, values and motivations) to consistently perform successfully within a specified domain (Vitello & Greatorex 2021:4). Participants cited that secondary caregivers lack knowledge of providing quality care to children and incompetency in performing the relevant skills such as bathing, feeding, changing positions, transferring children from bed to chair, performing simulations and talking to children because of limitations in their training curriculum, which does not give them a broader coverage of the skills. It is for this reason that it becomes difficult for secondary caregivers to provide quality care. Participants recommended training and demonstrations for caregivers to upskill and assist them in improving their knowledge and skills to render quality care. A study by Auberry (2018:26) found that secondary caregivers who did not receive formal education or practice experience regarding intellectual disabilities have inadequate knowledge and incompetency in providing quality care, hence the need for training and demonstrations. Zuurmond et al. (2019:47) affirmed that training and demonstrations assist caregivers to perform the specific skill with confidence and equip the caregivers with cognitive and psychomotor skills (Machalicek, Lang & Raulston 2015:112). Furthermore, demonstration enables a person to be competent in performing a skill. Dionne-Odom et al. (2017:2439) explained that skills in healthcare, such as position changing of a patient, require a specific technique that must be mastered before application. Therefore, secondary caregivers need to be demonstrated the skill because some of them did not receive formal training for such skill. Dionne-Odom et al. (2017:2440) emphasised that managers must optimise the preparedness and well-being of secondary caregivers, and this will promote self-esteem and confidence in secondary caregivers. The need for regular training and demonstrations is further affirmed by Narasimhan (2019:1688).

The second theme, stress reduction explains the necessity of working in a stress-free environment to be productive. Stress is an aversive stimulus people are exposed to, as a response they show in reaction to this stimulus, or as an interaction process, assuming that it is the lack of fit between the environment and the person, the notion that things are getting too much and out of balance (Hutmacher 2021:6). People are stressed out because of specific events or chronic pressures that place demands on them or threaten their well-being. Participants indicated that secondary caregivers are stressed by the work that they do. Secondary caregivers have a greater responsibility than other healthcare professionals, and hence, they become overwhelmed and stressed (Mbugua et al. 2021:3). Participants recommended the need for emotional support because, without emotional support, the psychological well-being of secondary caregivers may deteriorate, resulting in depression that can affect the quality of care given to children (Mbugua et al. 2021:3). Supervisors and colleagues must support secondary caregivers and must implement emotional support strategies such as listening to an employee’s concerns, allowing employees to talk about their emotions and delivering words of encouragement and guidance to help employees to regulate their emotions (Pohl et al. 2022:4). Strategies to reduce stress include providing emotional support, regular counselling, group therapies and parents’ involvement, which should be implemented by an employer. According to Sullivan and Miller (2015:8), part of the job of healthcare providers is to prepare caregivers for their emotions and reactions to the task, such as grieving for a loss of life, purpose and ability by building a network of resources. According to Bhoomadevi, Ganesh and Panchanatham (2021:426), emotional support will influence the confidence of secondary caregivers, thus reducing the burdens associated with caring. A study by Broglia et al. (2023:205) confirmed that counselling sessions are effective in improving depression, anxiety, well-being, hostility, social anxiety and stress. Group therapies are effective in dealing with stress and promote a sense of group cohesion and universality of experiences, because members share an understanding of the difficulties, they encounter and further feel that they are understood. When people with the same challenges meet and talk, an individual feels better because she or he can see that she or he is not alone (Acri et al. 2019:199). Learned techniques during group therapy sessions enhance the quality of care and relationships with clients, in this case, children with profound intellectual disabilities (Takalu, Hosseini & Khankey 2017:1). According to Sundal and Vatne (2020:7), to safeguard the child’s best interest, the collaboration between a healthcare professional and a parent is characterised by flexibility and reciprocity and by dialogues in action. Areas of collaboration are characterised by the healthcare professional’s sensitivity to the child’s reaction as well as by inputs from the parent. Parents depend on good interaction with the child, based on his or her knowledge of, and affiliation with the child.

The third theme, resources, emphasises the importance of resources in the workplace. Resource means a stock or supply of money, staff and other assets that an organisation can draw on to function effectively, and job resources are known to be key drivers of work engagement (Hakanen, Bakker & Turunen 2024:3). A shortage of such resources in health facilities is a barrier that may reduce access to health services and increase the risk of poor health outcomes (Qiu et al. 2022:3). The findings of the study revealed that secondary caregivers of children with profound intellectual disability experience a shortage of resources in the form of medical equipment and staff shortages (human resources). Medical equipment includes special wheelchairs, lifting machines, supporting cushions, linen, disposable nappies and special feeding utensils. Human resource shortages include inadequate staff, particularly caregivers. The shortage of human resources did impact negatively not only on the quality of patients’ care but also on the emotional well-being of caregivers. Reactions such as self-blame and guilt, feelings of discouragement, feelings of frustration and feelings of demotivation result (Moyimane, Matlala & Kekana 2017:2). Participants advised managers and policymakers to ensure that there is no scarcity of medical resources. Emmanuel et al. (2020:1) affirmed that it is the responsibility of managers to ensure that the working environment becomes conducive by providing with equipment required to render a service. Human resources also play a key role in the provision of quality care and the reduction in staff ‘burnout’, and shortage can lead to physiological exhaustion and unsatisfying work-life for those staff members who are on duty (Thor & Siegfried 2021:14). Managers must ensure that there is adequate staff and must strive to retain staff members because reasons for staff to quit jobs are found to be a low salary payment, a lack of appreciation and a high amount of work-related stress (Thor & Siegfried 2021:15).

The fourth theme, motivation, advocates that employees must be motivated to work. Employers must therefore put measures in place to motivate employees. Motivation is a process of stimulating people to action to accomplish goals. In the work goal context, the desire for money, success, recognition, job satisfaction and teamwork are the psychological factors stimulating people’s behaviour (Diem Vo, Tuliao & Chen 2022:49). Work motivation plays a vital role in the development of organisations, as it increases employee productivity and effectiveness. To achieve an organisation’s objectives, the employer depends on the performance of their employees. However, insufficiently motivated employees perform poorly. Employers, therefore, need their employees to work with complete motivation rather than just showing up at their workplaces (Diem Vo et al. 2022:49). Participants cited that secondary caregivers are not motivated to perform their duties because they are not well-remunerated and adequately recognised. The rationale for poor remuneration is not clearly understood but may be impacted by the lack of recognition of the caring process, the nature of the intellectual and developmental disability, and the lack of research on the level of knowledge caregivers have about intellectual disability. A study by Shi et al. (2022:630) emphasised that it is important to appreciate, reward and remunerate caregivers well. Participants made a plea for managers to motivate secondary caregivers through improved salaries, incentives, rewards and appreciation because they work very hard and long hours. Secondary caregivers receive a low salary that does not even cover their necessities such as food, rent, transport and providing for families. In comparison to other healthcare practitioners such as nurses, doctors, physiotherapists, social workers, occupational therapists, speech therapists and psychologists, secondary caregivers are not appreciated and are not getting incentives. The need to motivate caregivers is further supported by Auberry (2018:28). There is a need for a collective bargaining agreement that must go beyond wage rate, with provisions for retirement security, paid sick days, maternity leave, vacations and rational promotions to address the financial challenges of secondary caregivers (Hartz & Wright 2019:209). Appreciating, rewarding and providing incentives to secondary caregivers will not only motivate caregivers but will indirectly assist in dealing with staff shortages, thus easing the compatibility of a job and improving work-life balance (Chan et al. 2013:608).

### Limitations

The study was confined to one province of the country, thus making the perspectives of mental healthcare practitioners regarding how secondary caregivers of children with profound intellectual disabilities can be supported in other provinces unknown. Therefore, because of the qualitative nature of the study, the results of the study cannot be generalised to other provinces. However, the results of this study are likely to be applied to other settings in South Africa as the themes mentioned are universal issues and can therefore be used to improve the conditions of secondary caregivers nationally.

### Recommendations

From the results, the study recommends support for secondary caregivers. In supporting caregivers, it is recommended that perspectives of mental health care practitioners regarding how secondary caregivers of children with profound intellectual disabilities can be supported be considered and implemented. Future studies that will include secondary caregivers from other provinces are recommended. Recommendations are further discussed under the headings of recommendations for practice, policy development and research.

**Recommendations for practice:** A noticeable lack of support for secondary caregivers of children with profound intellectual disabilities continues to be a concerning issue. Regular training and demonstrations in the form of in-service training and workshops for secondary caregivers are recommended because it has been identified that the majority of secondary caregivers did not receive formal education or practice experience regarding intellectual disabilities and have inadequate knowledge about intellectual disability. Stress reduction for caregivers is recommended because it has been identified that caregivers are exposed to work-related stressors and stress, and managers are not providing support to caregivers, hence the plea for managers to provide emotional support and encourage and guide caregivers in the regulation of their emotions. Adequate resources are recommended because it has been found that there are inadequate resources in healthcare institutions for children with profound intellectual disabilities. Resources include human resources and equipment. Adequate resources would mean the need to employ more caregivers and other multidisciplinary team members such as social workers, occupational therapists, physiotherapists, medical practitioners and psychologists. Furthermore, managers would be expected to order medical equipment such as wheelchairs, lifting devices, machines and instruments that are used for children. It is recommended that caregivers be appreciated, rewarded and remunerated well because it has been identified that caregivers have stress, and the stressors are low salaries and lack of appreciation and rewards.

**Recommendations for policy development:** A review of policies on support of secondary caregivers of children with profound intellectual disabilities is recommended. The orientation policy needs to address the support strategy for employees with minimal or no experience in caring. Occupational health and safety policy should extensively give a direction on how managers must provide and maintain as far a reasonable and practical work environment. During the review, perspectives of mental healthcare practitioners regarding how secondary caregivers can be supported must be considered.

**Recommendations for nursing education:** It is recommended that education and training curricula for secondary caregivers of children with profound intellectual disabilities be reviewed. The narrow outcomes of the curriculum should be broadened to include outcomes such as assessment, feeding, dressing, lifting, position changing and stimulations of a child with intellectual disability because it is evident that including such outcomes will ensure that caregivers become knowledgeable about intellectual disability and equipped to perform skills necessary.

**Recommendations for future studies:** Future studies that will include secondary caregivers of other provinces of the country are recommended because the study was not inclusive of secondary caregivers in other provinces. Furthermore, future studies that will explore the perspectives and experiences of primary caregivers, secondary caregivers and managers of care institutions for children with profound intellectual disabilities are recommended because, currently, their perspectives and experiences are unknown. A similar study utilising a mixed method is recommended for better triangulation.

## Conclusion

Secondary caregivers are principal carers of children with profound intellectual disabilities in healthcare facilities. The work that they do is demanding and overwhelming, and hence, secondary caregivers require support. The study explored and described the perspectives of mental healthcare practitioners regarding the conditions under which secondary caregivers of children with profound intellectual disabilities work and how can they be supported. Knowledge and skills development, stress reduction, resources and motivation were identified as perspectives of mental healthcare practitioners on how secondary caregivers can be supported. Perspectives, if applied, can enormously reduce burdens experienced by secondary caregivers while caring for children. Perspectives can further be applied in future studies aiming to develop programmes, frameworks and intervention strategies aiming to support secondary caregivers.
